# High-Throughput and Automated Acoustic Trapping of Extracellular Vesicles to Identify microRNAs With Diagnostic Potential for Prostate Cancer

**DOI:** 10.3389/fonc.2021.631021

**Published:** 2021-03-25

**Authors:** Anson Ku, Jacob Fredsøe, Karina D. Sørensen, Michael Borre, Mikael Evander, Thomas Laurell, Hans Lilja, Yvonne Ceder

**Affiliations:** ^1^ Department of Translational Medicine, Lund University, Malmö, Sweden; ^2^ Department of Molecular Medicine, Aarhus University Hospital, Aarhus, Denmark & Department of Clinical Medicine, Aarhus University, Aarhus, Denmark; ^3^ Department of Urology, Aarhus University Hospital, Aarhus, Denmark & Department of Clinical Medicine, Aarhus University, Aarhus, Denmark; ^4^ Department of Biomedical Engineering, Lund University, Lund, Sweden; ^5^ Urology Service, Department of Surgery, Memorial Sloan Kettering Cancer Center, New York, NY, United States; ^6^ Genitourinary Oncology Service, Department of Medicine, Memorial Sloan Kettering Cancer Center, New York, NY, United States; ^7^ Department of Laboratory Medicine, Memorial Sloan Kettering Cancer Center, New York, NY, United States; ^8^ Department of Laboratory Medicine, Lund University, Lund, Sweden

**Keywords:** acoustic trapping, extracellular vesicles, prostate cancer, liquid biopsy, microRNA, ncRNA

## Abstract

Molecular profiling of extracellular vesicles (EVs) offers novel opportunities for diagnostic applications, but the current major obstacle for clinical translation is the lack of efficient, robust, and reproducible isolation methods. To bridge that gap, we developed a microfluidic, non-contact, and low-input volume compatible acoustic trapping technology for EV isolation that enabled downstream small RNA sequencing. In the current study, we have further automated the acoustic microfluidics-based EV enrichment technique that enables us to serially process 32 clinical samples per run. We utilized the system to enrich EVs from urine collected as the first morning void from 207 men referred to 10-core prostate biopsy performed the same day. Using automated acoustic trapping, we successfully enriched EVs from 199/207 samples (96%). After RNA extraction, size selection, and library preparation, a total of 173/199 samples (87%) provided sufficient materials for next-generation sequencing that generated an average of 2 × 10^6^ reads per sample mapping to the human reference genome. The predominant RNA species identified were fragments of long RNAs such as protein coding and retained introns, whereas small RNAs such as microRNAs (miRNA) accounted for less than 1% of the reads suggesting that partially degraded long RNAs out-competed miRNAs during sequencing. We found that the expression of six miRNAs was significantly different (P_adj_ < 0.05) in EVs isolated from patients found to have high grade prostate cancer [ISUP 2005 Grade Group (GG) 4 or higher] compared to those with GG3 or lower, including those with no evidence of prostate cancer at biopsy. These included miR-23b-3p, miR-27a-3p, and miR-27b-3p showing higher expression in patients with GG4 or high grade prostate cancer, whereas miR-1-3p, miR-10a-5p, and miR-423-3p had lower expression in the GG4 PCa cases. Cross referencing our differentially expressed miRNAs to two large prostate cancer datasets revealed that the putative tumor suppressors miR-1, miR-23b, and miR-27a are consistently deregulated in prostate cancer. Taken together, this is the first time that our automated microfluidic EV enrichment technique has been found to be capable of enriching EVs on a large scale from 900 μl of urine for small RNA sequencing in a robust and disease discriminatory manner.

## Introduction

Extracellular vesicles (EVs) are 50–1,000 nm membrane-encapsulated particles that are secreted by outward budding or fusion of the multi-vesicular endosome with the plasma membrane. EVs contain various types of biomolecules, *e.g.* protein, lipids, and nucleic acids, that reflect the cell of origin and can be found in all biological fluids including blood, cerebral-spinal fluid, semen, urine, saliva, and breast milk (1). In pathological conditions like cancer, EVs have been shown to facilitate disease progression. Due to their reported disease-specific content and accessibility, EVs have been proposed as a potential new, non-invasive source of biomarkers during routine liquid biopsy (2). However, critical requirements for the introduction of EVs as a routine clinical diagnostic entity include the development of EV isolation and analytical methods manifesting efficient, robust, reproducible, high-throughput performance, and ease of use. Currently, ultracentrifugation with or without cushion gradient remains the standard isolation technique. However, the use of ultracentrifugation in a clinical setting is hampered by the labor, volume, and time requirement. There are a number of commercial and published methods for EV enrichment such as size-exclusion separation (qEV), polymer precipitation (Exoquick), membrane filtration, affinity-based purification, and microfluidics based separation, but many are not suitable for clinical translation due to long incubation time (Exoquick) or cost (immuno-affinity based purification) (3, 4). The number of automated EV isolation methods remains few (qEV, membrane filtration, iDEP), has yet to be tested on large scale, or is not low sample-volume compatible (3, 5–8). We have developed a non-contact and automated enrichment technology termed “acoustic trapping” that operates on the principle that particles in suspension will interact when exposed to ultrasound in a manner that results in their aggregation and retention against fluid flow (9). We have previously shown that the acoustic trapping technology can enrich EVs of all sizes from biological samples such as plasma or urine using a half-wavelength acoustic resonator setup (10, 11). In addition, we have utilized an optimized pipeline to interrogate miRNA expression from urinary EVs by next-generation sequencing (12).

The notion of overdiagnosis and overtreatment of prostate cancer (PCa) is gaining recognition, as indolent forms that never impact longevity or quality of life are found to increase with age (13). Hence, a major challenge is the selective detection and management of lethal forms of PCa at very early and curable stages. A supplemental biomarker that can stratify patient with likely aggressive PCa is urgently needed as current criteria to determine who should receive surveillance are imprecise leading to delay in treatment of aggressive PCa requiring immediate intervention and overtreatment of men who do not benefit from curative treatment. In that regard, urinary EVs have shown early promise as a non-invasive source of biomarkers. The contribution of EVs released into the urethra from the prostate gland *via* the prostatic duct is supported by the presence of prostate specific markers such as TMPRSS2:ERG fusion and PCA3 in urinary EVs (14, 15). In-addition, miRNA obtained from urinary EVs of men with PCa have demonstrated prognostic performance (16, 17). Aiming to reduce overdiagnosis and overtreatment, we leveraged our previous EV isolation work based on access to urine samples with clinical and histopathologic data from a well-annotated prostate biopsy cohort to identify miRNA biomarkers from urinary EVs that can stratify patients likely to have high grade PCa from those harboring low grade PCa.

## Materials and Methods

### Patient Cohort

The patient cohort consists of first void morning urine from 207 men who, on the same day, were subjected to transrectal ultrasound-guided biopsy of the prostate due to suspicion of PCa. All patients were previously biopsy (Bx) naïve; the majority had ≥10 Bx cores where samples from 60 patients showed no histopathologic evidence of cancer in any of the needles, and 147 were PCa-positive Bx, *i.e.* PCa present in at least one needle. Forty-six of the PCa-positive samples had Gleason score greater than or equal to eight (ISUP Grade Group [GG] four or higher). Prior to the same day appointment for biopsy, patients collected urine at home in a 50 ml Falcon tube with a Stabilur™ tablet. The urine was centrifuged at 500 × g for 5 min with the supernatant being transferred to a new tube and cryopreserved at −80^°^C at the Department of Urology, Aarhus University Hospital, Denmark and later shipped on dry ice to Lund University, Sweden. Cohort data are summarized in **Table 1** and **Supplemental Table 1** with additional details documented in Fredsoe et al. as cohort 4 (18). Upon arrival and prior to processing, sample order was randomized to minimize processing bias. In addition, the investigators were blinded to the clinical and histopathological status of all patient samples until next generation sequencing had been completed.

**Table 1:** Patient characteristics showing median age, serum PSA, pre-biopsy digital rectal examination (DRE) status, clinical stage, Gleason grade from biopsy and number of positive needles stratified as biopsy-positive (Bx-positive) *versus* Bx-negative groups or as Gleason grade ≥ 4 *versus* Gleason grade ≤ 3.

	Bx-positive, n = 147	Bx-negative, n = 60	Gleason grade ≥ 4, n = 60	Gleason grade ≤ 3, n = 147
**Median Age (range)**	68.8 (43.1–79.8)	65.1 (47.6–79.8)	69.6 (52.2–79.3)	67.0 (43.1–79.8)
**Serum PSA levels, n (%)**				
≤10 ng/ml	76 (51%)	42 (70%)	42 (70%)	76 (51%)
>10 ng/ml	73 (49%)	18 (30%)	18 (30%)	73 (49%)
Unknown				
Median PSA, ng/ml (range)	9.1 (1.9–465.9)	7.7 (3.1–54.7)	11.3 (4.6–465.9)	7.6 (1.9–46.8)
**Pre-biopsy DRE status, n (%)**				
Positive	88 (59.1%)	11 (18.3%)	46 (75.4%)	53 (35.8%)
Negative	54 (36.2%)	39 (65%)	12 (19.7%)	81 (54.7%)
Unknown	0 (0%)	2 (3.3%)	0 (0%)	2 (1.4%)
Uncertain	7 (4.7%)	8 (13.3%)	3 (4.9%)	12 (8.1%)
**T-stage, n (%)**				
cT1	46 (30.9%)	NA	9 (15.0%)	37 (25.2%)
cT2	43 (28.9%)	NA	20 (33.3%)	24 (16.3%)
cT3	48 (32.2%)	NA	28 (46.7%)	17 (11.6%)
unknown	12 (8.1%)	NA	3 (5%)	9 (6.1%)
**Gleason grade, n (%)**				
1	31 (20.8%)	NA	0 (0%)	31 (21.1%)
2	43 (28.9%)	NA	0 (0%)	42 (28.6%)
3	13 (8.7%)	NA	0 (0%)	14 (8.8%)
4	28 (18.8%)	NA	26 (43.3%)	0 (0%)
5	34 (22.8%)	NA	34 (56.7%)	0 (0%)
Unknown	0 (0%)	NA	NA	60 (40.8%)
**Gleason grade, n (%)**				
≤ 3	101 (31.2%)	NA	0 (0%)	147 (100%)
≥ 4	46 (68.8%)	NA	60 (100%)	0 (0%)
**Positive needles, n (%)**				
0	0 (0%)	60 (100%)	0 (0%)	60 (39%)
1	30 (20.1%)	0 (0%)	5 (10%)	25 (16%)
2	22 (14.8%)	0 (0%)	4 (8%)	18 (12%)
3	15 (10.1%)	0 (0%)	4 (8%)	11 (7%)
4	18 (12.1%)	0 (0%)	4 (8%)	13 (8%)
5	19 (12.8%)	0 (0%)	10 (19%)	9 (6%)
6	8 (5.4%)	0 (0%)	4 (8%)	4 (3%)
7	6 (4%)	0 (0%)	3 (6%)	2 (1%)
8	7 (4.7%)	0 (0%)	5 (10%)	2 (1%)
9	10 (6.7%)	0 (0%)	3 (6%)	8 (5%)
10	14 (9.4%)	0 (0%)	10 (19%)	3 (2%)

The median age of the Bx-positive and Bx-negative groups is 68.8 and 65.1, respectively. The serum PSA levels are 9.1 and 7.7, respectively. Non-parametric testing revealed significant difference between the age and PSA level of two cohorts (P < 0.0001 and P = 0.0012, respectively). Brackets contain minimum and maximum of the related parameter or percentage of population.

### Sample Processing and EV Enrichment

**Figure 1:**
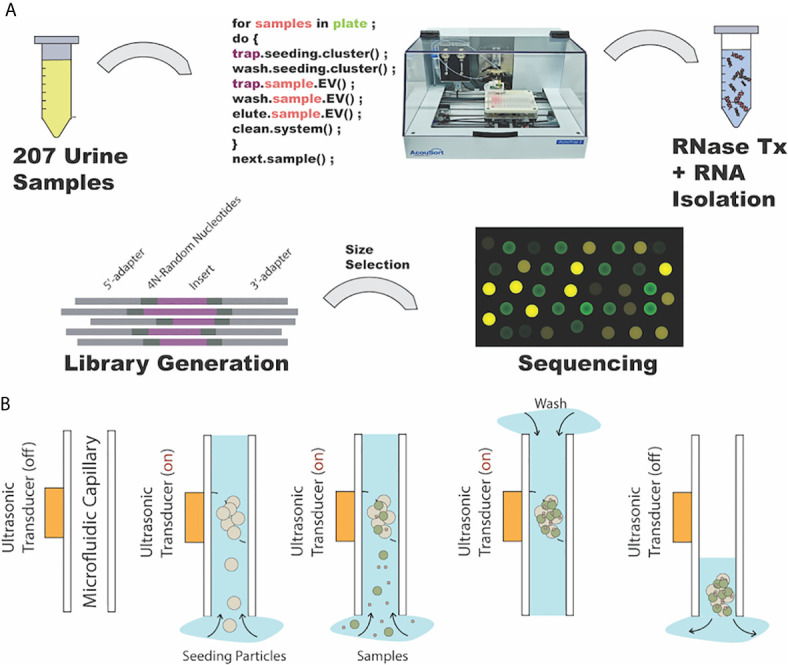
Urinary extracellular vesicle (EV) isolation and processing workflow. **(A)** Schematic of workflow beginning with urine sample randomization, automated EV isolation by AcouTrap, RNA isolation, small RNA library preparation and finally sequencing. **(B)** Illustration of acoustic trapping steps. From left to right, ultrasonic transducer coupled to microfluidic channel where sample flows. Seed particles (12 μm) that will serve as anchors for vesicles are trapped. Next, samples containing vesicles are aspirated through the seed cluster where EV enrichment occurs. After sample aspiration, seed cluster with enriched EVs is washed with PBS and lastly dispensed into designated well.

Urine samples of 1 ml were processed as illustrated in **Figure 1A**. Briefly, undiluted cryopreserved urine was equilibrated to room temperature and centrifuged at 2,000 × g for 10 min to remove cells and cellular debris prior to acoustic enrichment. Next, patient samples were split into two 450 µl fractions and aliquoted into a 96-well plate for EV isolation by acoustic trapping. A script containing the operating parameters such as ultrasonic frequency (4.2 Mhz), peak-to-peak voltage (10 V_pp_), flow rate (15 μl/min), total volume acquisition (900 μl total trapped in two equal fractions), and sample location was used to automate the AcouTrap system (AcouSort AB, Sweden). Next, the ultrasonic transducer was powered to trap 12 µm seed particles followed by washing to remove excess particles (**Figure 1B** **)**. Urinary EV enrichment was performed on the trapped seed particles cluster followed by washing and elution in 60 µl of PBS. After trapping, two processed fractions from the same sample were pooled and stored at −80°C until RNA isolation. Additional samples were split and loaded onto new wells of the 96-well plate such that acoustic trap can continuously process the added urine samples until the capacity of the 96-well plate, 32 samples, is reached. To ensure the performance of the AcouTrap, quality control was performed daily by trapping 290 nm polystyrene beads (Bangs Laboratory). A performance of 10% trapping efficiency or above was accepted.

### RNA Isolation and Library Preparation

Enriched EVs were thawed on ice and treated with 10 μg/ml of RNase A at 37°C to remove free circulating RNA. As a positive control for the RNase activity treatment, total RNA isolated from PC3 cell line was also treated in parallel with RNase A. Next, total RNA was isolated using single cell RNA isolation kit (Norgen) per manufacturer’s protocol with β-mercaptoethanol added during the lysis step. RNA eluted from the column was transferred immediately for small library preparation using NEXTFlex library preparation kit (Perkin-Elmer, USA) using a minor modification of the manufacturer’s protocol. Briefly, 10.5 µl of eluent from each sample was ligated with the 3′adapter overnight at 16°C. Excess adapters were removed and inactivated followed by 5′adapter ligation, cDNA synthesis, and 28 cycles of PCR amplification. Libraries were cleaned and size-selected (approximately 140–160 bp) by BluePippin system using 3% agarose gel cartridge (Sage Science, USA). We used the Qubit DNA HS assay (Thermo Scientific, USA) and Bioanalyzer DNA HS chip (Agilent, USA) to quantify cDNA concentration and size, respectively. cDNA libraries were pooled and sequenced on the NextSeq 550 system (Illumina, USA) using five high-output 500/550 flow cell single-end, 75 bp reads.

### Data Processing and Analysis

Sequencing results were de-multiplexed using bcl2fastq (version v2.20.0.422). The 3′adapter was trimmed and sequences less than 15 nt in length were removed using cutadapt per NEXTFlex manufacturer’s instruction. Additional filtering and mapping were performed using ExceRpt, a previously reported pipeline (10). Briefly, low-quality, low-complexity reads and contaminants were removed. The remaining reads were mapped to human reference genome (hg38) with one-mismatch using STAR alignment software yielding small RNA counts not including those mapped to rRNAs. Differential expression analysis was performed using DESeq2 on samples grouped into Gleason high (GG4 and GG5) compared to Gleason low (Bx-negative and GG1–GG3) with batch effect accounted for. Differentially expressed genes are filtered based on the criteria of log2 fold change of less than −1 or greater than 1 and an adjusted P-value of less than 0.05 (see **Supplemental Method** for Rscript) (19). All downstream data analysis was performed using R statistical environment version 3.4.3 and RStudio version 1.2.5001.

## Results

### Patient Characteristics and Sample Collection

The patient cohort consisted of urine samples from 60 men with ≥GG4 and 147 men with ≤GG3 or biopsy-negative (collective denoted as ≤GG3) (**Table 1**). The median age of the ≥GG4 and ≤GG3 were 68.8 (Interquartile range, IQR: 64.3–72.9 years) and 64.8 years (IQR 58.7–69.2 years), respectively, at the time of biopsy, and the median serum PSA levels were 9.1 (IQR 6.1–18.6 ng/ml) and 7.0 ng/ml (IQR 5.3–10.6 ng/ml), respectively. Comparison of the two group’s age and serum PSA level showed significant difference by non-parametric Mann–Whitney (P = 0.004 and P = 0.004, respectively).

### EV Enrichment, RNA Isolation, and Library Preparation

**Figure 2:**
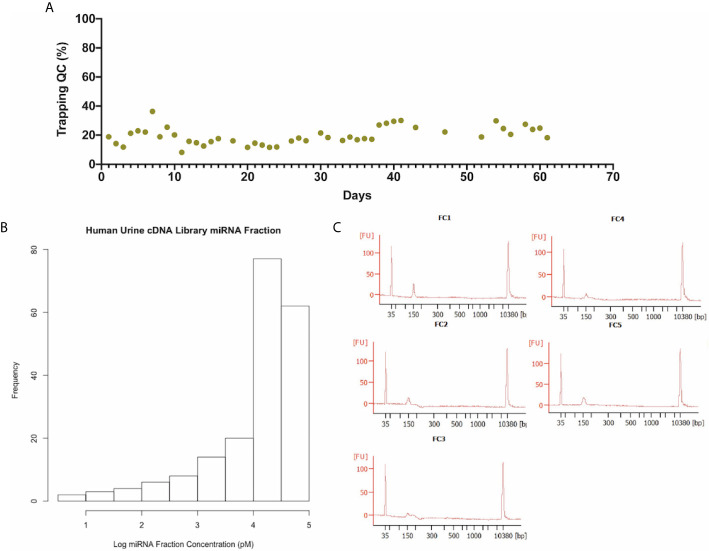
Extracellular vesicle isolation and library preparation. **(A)** Quality control performed daily on the AcouTrap with 290nm polystyrene beads illustrating the stability of the instrument. The trapping efficiency’s mean and standard deviation were 19.7 ± 6% with C.V. of 31%. **(B)** Histogram of small RNA library concentration between sizes of 140–160 nt as quantified by Bioanlyzer. The median concentration of the samples is 11 nM and an interquartile range of 29 nM. **(C)** cDNA library profile after sample pooling and size selection. Fluorescent peak around 151 nt corresponding to inserts of approximately 20 nt in length flanked by 5′ and 3′adapters. The final concentrations of the pools, noted as FC1–FC5, are 0.52, 0.56, 0.21, 0.23, and 0.6 nM between 140 and 160 nt.

Prior to EV enrichment from urine samples, trapping assessment was performed on the AcouTrap using 290 nm polystyrene beads to ensure proper function (**Figure 2A**). Over the period of operation, the trapping efficiencies were 19.7 ± 6% (mean ± s.d.) with a coefficient of variation of ~30%. Analysis of the trapping variations revealed that it is a result of using three different acoustic trapping units throughout the experiment (data not shown). Overall, a total of 199 out of the 207 samples (96%) were successfully enriched by the acoustic trap. Eight samples (six Bx-positive and two Bx-negative) failed due to the presence of air bubbles in the resonant cavity or seed cluster washout during trapping. Statistical testing revealed that trapping failures were homogeneously distributed between samples with ≥GG4 and ≤GG3 (P > 0.05, Wilcoxon Signed-Rank Test). Library preparation from total RNA resulted in a total of 173 (87%) of the 199 samples with quantifiable cDNA by Bioanalyzer (**Figure 2B**). The resulting 173 samples consisted of 46 patients with ≥GG4 PCa and 127 patients with ≤GG3 PCa as well as Bx-negative samples. The median age of patients with ≥GG4 and ≤GG3 was 68.8 (IQR 64.5–73.1 years) and 66.3 years (IQR 60.8–70.5 years) and the PSA levels were 11.3 ng/ml (IQR 6.8–28.6 ng/ml) and 7.7 ng/ml (IQR 5.9–14 ng/ml), respectively. Non-parametric analysis by Mann–Whitey revealed significant difference in age and PSA level between patients with ≥GG4 and ≤GG3 (P = 0.014 and P = 0.006, respectively).

The first-quartile, median, and third-quartile concentrations of the prepared sample within 140–160 nt (corresponding to approximately 10–30 nt inserts) were 11, 21, and 40 nM, respectively, with an interquartile range of 29 nM. Pooling of the libraries into five aliquots followed by size selection resulted in peaks ranging from 149 to 152 bp for the five aliquots (**Figure 2C**). The peak sizes corresponded to inserts of 19–22 nt in length. The final concentrations of the pools (FC1–FC5) were 0.52, 0.56, 0.21, 0.23, and 0.6 nM in the size range of 140–160 nt.

### Sequencing Results

**Figure 3:**
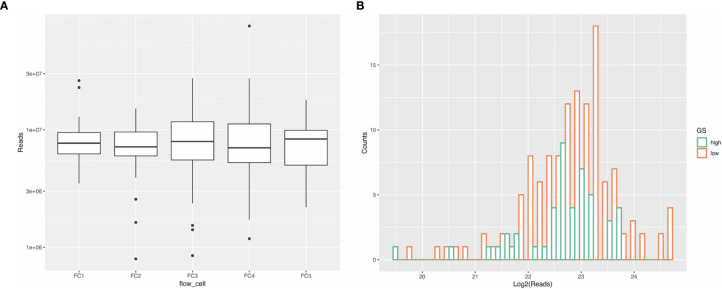
Sequencing results. **(A)** Sequencing with five high-output flow cells yielded a total of 301 × 10^6^, 352 × 10^6^, 398 × 10^6^, 388 × 10^6^, and 371 × 10^6^ clusters passed filtering. The median reads of the five flow cells range from 7 × 0^6^ to 8 × 10^6^. **(B)** The read distribution between sample groups with Gleason grade ≥4 (green) and **≤**3 (orange) is not statistically different by Mann–Whitney non-parametric analysis (P > 0.05).

**Figure 4:**
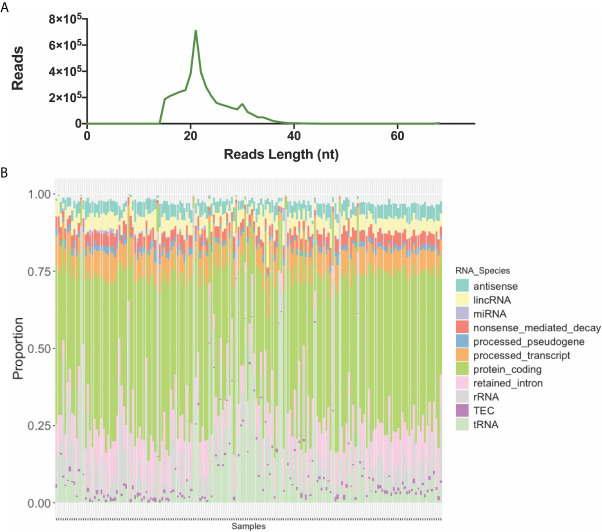
Aligned reads. **(A)** The average length of the mapped reads across sample are 21 nt in length. **(B)** The RNA species mapped to the human genome showed predominantly protein coding (green), tRNAs (purple), retained intron (pink), processed transcript (blue), and nonsense-mediated decay RNAs (red).

**Figure 5:**
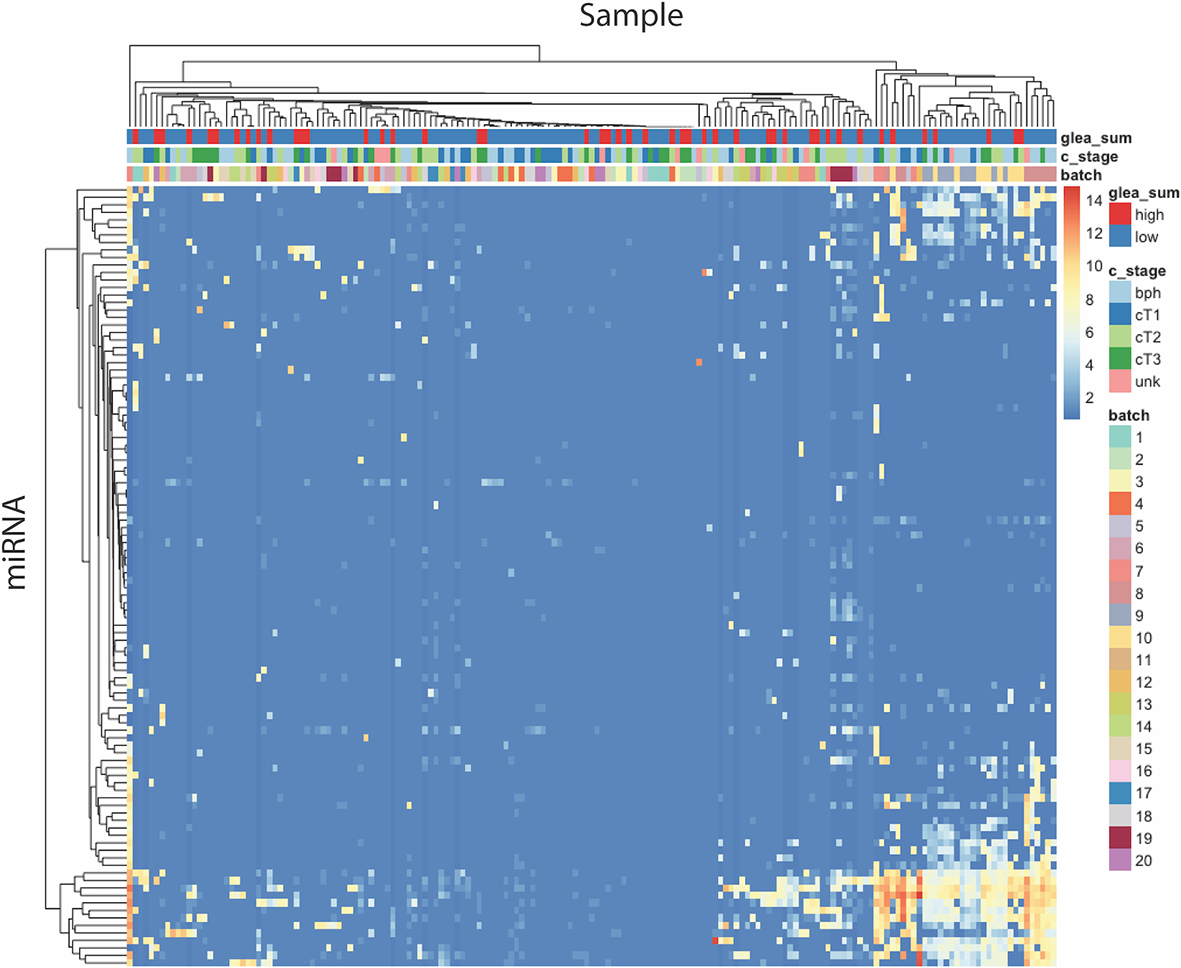
Unsupervised clustering of miRNAs expression revealed no discernible patterns between clinical parameters and miRNAs.

The sequencing of the 173 samples in five high-output flow cells resulted in a total of 1.5 × 10^9^ reads with 301 to 398 × 10^6^ passing clusters per cell, and 7.7 × 10^6^, 7.1 × 10^6^, 8.0 × 10^6^, 7.0 to 8.4 × 10^6^ median reads for each flow cell (**Figure 3A**). To determine if differences existed in the distribution of reads (before alignment) between ≥GG4 groups and ≤GG3, we compared the two by Mann–Whitney non-parametric analysis and found that they were not significantly different (**Figure 3B**). The number of mappable reads ranged between 9 × 10^4^ and 2 × 10^7^. Analysis of the mapped RNA species revealed that the average length of the reads across samples was 21 nt in length (**Figure 4A**), which was expected as the libraries were size selected for inserts ~10–30 nt long. Contrary to our expectations, we found that a total of 80% of the mapped reads were derived from long RNAs such as protein coding, retained intron, processed transcript, lincRNA, antisense, and nonsense-mediated decay (**Figure 4B** and **Supplemental Table 2**), whereas on average, 4 × 10^3^ reads per sample were mapped to miRNAs. The seven most abundant miRNAs found were let-7f-5p, let-7b-5p, miR-30d-5p, let-7a-5p, miR-375, miR-92a-3p, and miR-21-5p (**Supplemental Table 3**). The data have been deposited in NCBI’s Gene Expression Omnibus (Edgar et al., 2002) and are accessible through GEO (Data Availability Statement). To determine if the miRNA expressions in our dataset reflect the underlying clinical parameters, we performed unsupervised hierarchical clustering, but the samples did not cluster into discernible groups (**Figure 5**). In order to ascertain the validity of the miRNA expression profile derived from urinary EVs, we analyzed our results to the miRNA expression reported by Cheng et al. by significant correlation by linear regression (P < 0.05, **Supplemental Figure 2**).

**Figure 6:**
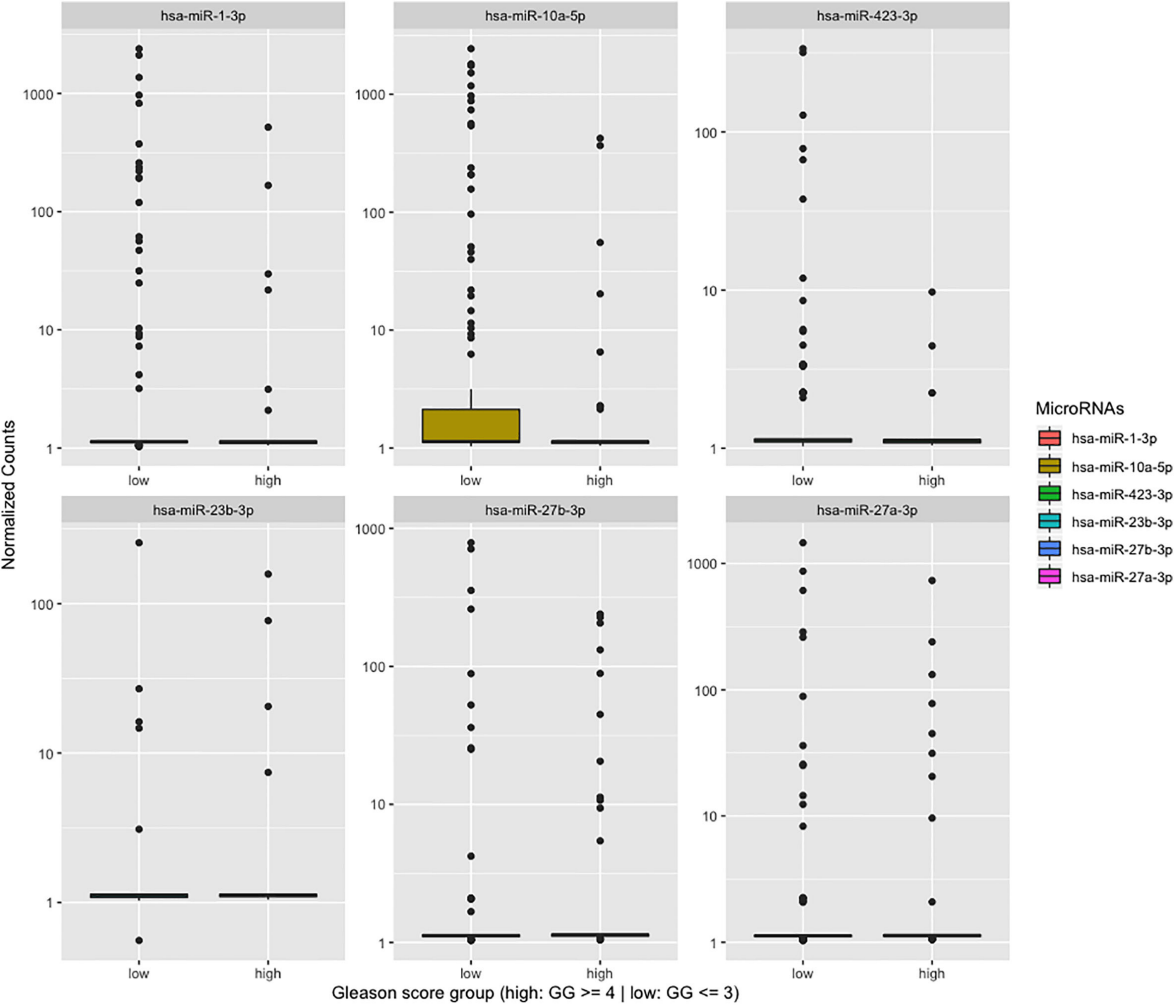
Boxplot showing differentially expressed miRNAs between two groups, Gleason grade 4 (≥GG4: high) and ≤GG3 with Bx-negative (low).

To better address the question of whether miRNA expression can be used to stratify patients with high risk tumor, we performed differential expression (DE) analysis comparing miRNA profiles from patients with high (≥GG4, n = 46) *vs*. low GG and Bx-negative samples (≤GG3 and Bx-negative, n = 127). The results yielded a total of 14 significantly deregulated miRNAs after adjustment for false discovery rate (P_adj_ < 0.05) (**Figure 6** and **Table 2**). However, inspection of the normalized counts of miR-183, -100, -205, -223, -615, -29a, -99b, and -4433a revealed that their differential expression is likely due to a limited number of samples containing detectable reads that artificially inflated their aggregate expression in the ≥GG4 group. Thus, their presence in our differential expression analysis could be type 1 statistical error caused by the low number of reads in our samples and should be interpreted with caution. By contrast, the expression of miR-23b-3p, miR-27a-3p, miR-27b-3p, miR-1-3p, miR-10a-5p, and miR-423-3p contained detected reads in sufficient samples. In addition, we have compared the urinary EVs’ miRNA expression profile of Bx-positive and Bx-negative patients but did not observe any differentially expressed miRNAs.

**Table 2:** Table of differentially expressed miRNA comparing Gleason score ≥8 to the Gleason score ≤7 and Bx-negatives adjusted for batch effect.

miRs	baseMean	log2FoldChange	lfcSE	stat	p value	padj
hsa-miR-1-3p	60.29	−1.76	0.49	−3.60	0.0003	0.0037
hsa-miR-10a-5p	84.26	−1.30	0.44	−2.95	0.0032	0.0239
hsa-miR-423-3p	7.03	−0.99	0.35	−2.83	0.0046	0.0271
hsa-miR-183-5p	1.37	0.75	0.26	2.89	0.0039	0.0247
hsa-miR-100-5p	1.48	0.84	0.26	3.19	0.0014	0.0136
hsa-miR-205-5p	4.70	0.88	0.30	2.91	0.0036	0.0246
hsa-miR-223-3p	2.99	0.89	0.30	3.00	0.0027	0.0223
hsa-miR-615-3p	1.94	1.04	0.28	3.69	0.0002	0.0033
hsa-miR-23b-3p	2.92	1.09	0.30	3.67	0.0002	0.0033
hsa-miR-99b-5p	2.95	1.15	0.29	4.01	0.0001	0.0017
hsa-miR-27b-3p	20.31	1.30	0.41	3.18	0.0015	0.0136
hsa-miR-27a-3p	29.86	1.51	0.39	3.83	0.0001	0.0027
hsa-miR-4433a-3p	6.02	1.53	0.31	4.96	0.0000	0.0000
hsa-miR-29a-3p	9.33	1.56	0.29	5.31	0.0000	0.0000

**Figure 7:**
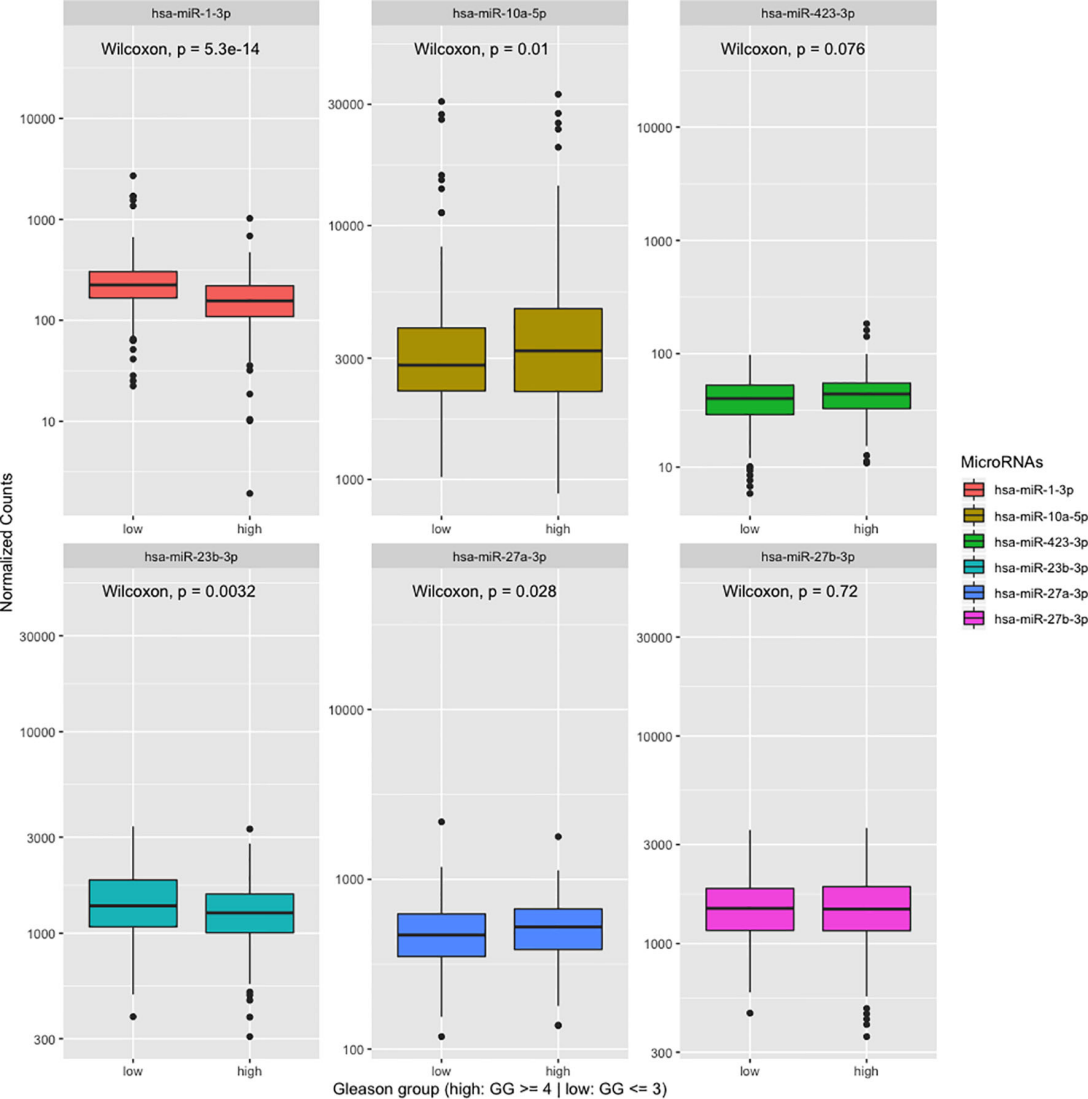
Expression of the differentially expressed microRNAs found in our analysis in the TCGA dataset grouped by Gleason grade 4 (GG4 or greater: high) *versus* GG3 or lower and Bx-negative (low) n = 497.

**Figure 8:**
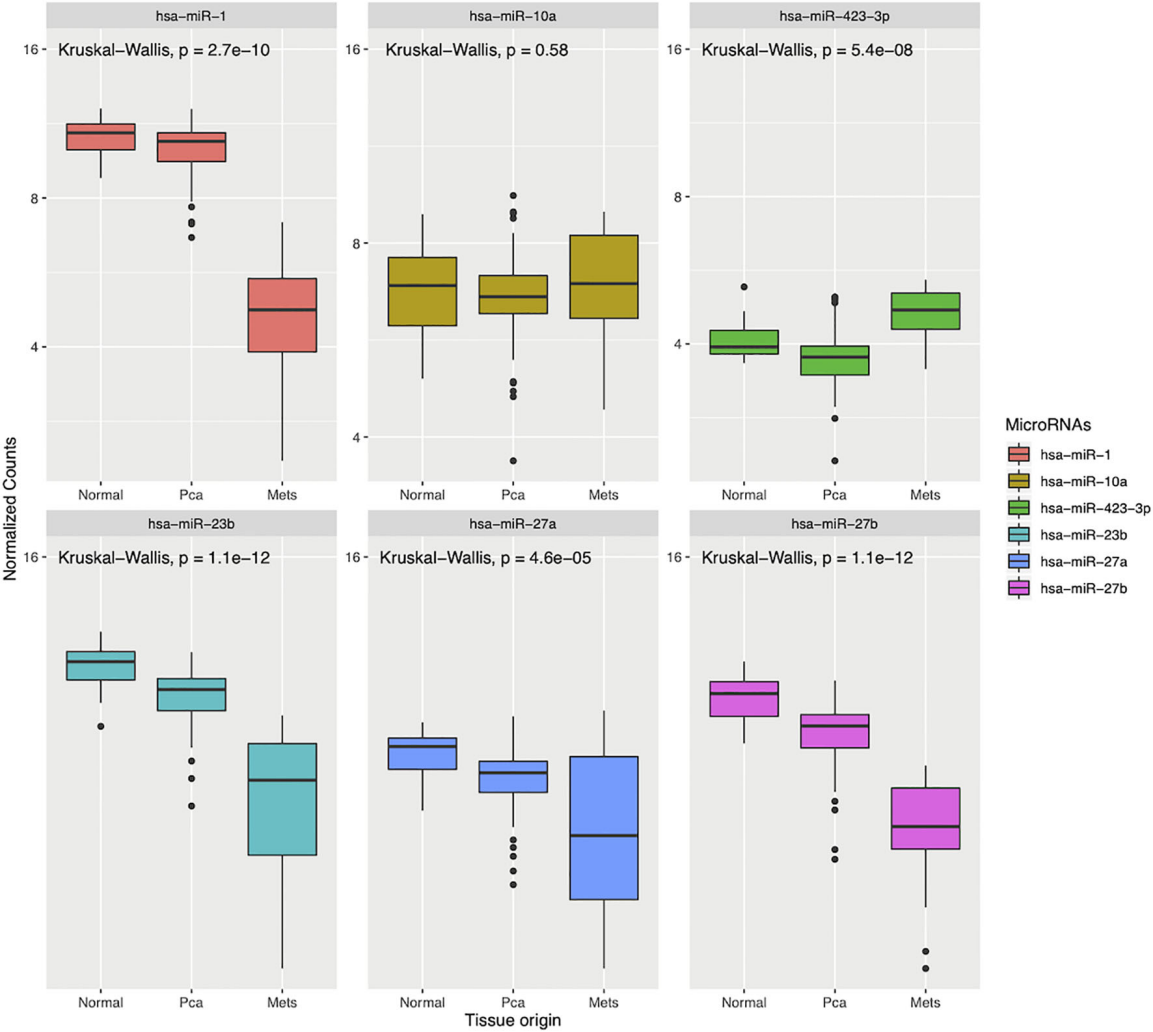
Expression of the differentially expressed microRNAs found in our analysis in the Taylor et al. dataset grouped by normal, prostate cancer (PCa) and metastatic samples (Mets), n = 141.

In order to explore the possibility that our differentially expressed miRNAs are also deregulated in clinical primary PCa, we analyzed the expression level of our six curated miRNAs from above in the TCGA prostate dataset (prad, n = 497). We found that four out of the six miRNAs are significantly deregulated by Wilcoxon test (P < 0.05) with miR-10a and miR-27a enriched while miR-1 and miR-23b are down-regulated in the ≥GG4 group (**Figure 7**). In a similar manner, we investigated our six miRNAs in the Taylor et al. dataset, which contains normal, primary, and metastatic PCa samples (n = 141) (20). Our analysis revealed that five of the six miRNAs are deregulated with miR-423 enriched in the metastatic samples while miR-1, -23b, -27a, and -27b are significantly down-regulated in the PCa and metastatic samples by Kruskal–Wallis test (P < 0.05) (**Figure 8**). Next, we examined the concordance between the miRNA expression in both public datasets and found that miR-1 and miR-23b are consistently down-regulated in high grade PCa, *i.e*. high GG or in PCa and metastatic samples, while miR-27a is enriched in the ≥GG4 group but down-regulated in the PCa and metastatic samples. Since miR-1 is co-transcribed with miR-133a and miR-133b, we hypothesize that miR-133a and miR-133b would also be down-regulated along with miR-1. Indeed, miR-133a and miR-133b expressions are lower in high risk PCa compared to low risk PCa (**Supplemental Figure 1**). Interestingly, we found that miR-23b is down-regulated in tissue samples from TCGA and Taylor dataset but are up-regulated in our urine analysis, whereas miR-1 is consistently down-regulated in all three analyses.

## Discussion

The past decade has unraveled a role of EVs as key players in disease progression. Thus, translation of EV content for diagnostic purposes could provide additional information for clinical management of diseases including PCa. To that end, we have shown that acoustic trapping can be scaled up for high throughput isolation of urinary EVs from patients with or suspected of PCa. We have demonstrated that our automated acoustofluidic EV isolation system, the AcouTrap, is a robust EV isolation method with less than five percent failure rates out of 207 samples. The two main mode of failures were due to bubble formation and seed cluster washout. These problems could be remediated by ensuring that temperature of the samples was sufficiently equilibrated in order to prevent bubble formation and increasing the number of fractions trapped per sample in order to reduce seed cluster washout.

At this time, it is difficult to compare the performance of our automated EV enrichment method to the published PCa EV biomarker studies that relied on hydrostatic filtration dialysis (21), differential ultracentrifugation (22, 23), or chemical precipitation (24) for enriching urinary EVs due to the dearth of information regarding their success rates and processing time. With our current instrument setup, the processing time of each sample was under 2 h with little manual labor other than dispensing the samples onto and recovering the eluent off of a 96-well plate. It is expected that with improvements to the design of the instrumentation such as the dimension of the acoustic resonator, it is possible to achieve even lower processing time thus enabling translation of EVs as a future clinical diagnostic entity. To our knowledge, this is the first time an automatic microfluidic EV isolation technique was utilized to enrich urinary EVs from clinical samples on this scale.

Harnessing urinary EVs for biomarkers could enable convenient and non-invasive approach to discriminate patients with high risk PCa. Currently, the Gleason grade is one of the best prognostic indictors for PCa risk based on the cellular differentiation pattern of PCa from biopsied materials. Studies have shown that Gleason grade group provides significant predictive power for 5-year biochemical recurrence (BCR), *i.e.* rising PSA after radical prostatectomy, with increasing Gleason grade group positively correlating with BCR recurrence (25, 26). Therefore, we dichotomized our patient cohort into ≥GG4 and ≤GG3 or high and low risk groups in order to determine if miRNAs from urinary EVs can differentiate the patients with high likelihood of BCR. Towards that end, we performed small RNA-sequencing on 173 urinary EV samples in order to identify potential biomarkers that can stratify patients with high or low GG. To our surprise, a large percentage of the mapped reads corresponded to protein coding RNA, lincRNA, and nonsense-mediated decay that are normally hundreds to thousands of base-pair in length. Their large representation in our reads suggested that they have been degraded during either collection or later freezing and thawing (27, 28). As a result, the degraded RNA fragments out-competed the miRNAs during cluster formation in the sequencing flow cell, thereby reducing the number of miRNA reads. In comparison, our previous work performing acoustic trapping on fresh urine samples treated with DTT showed that miRNA composed greater than 10% of the reads, which is in agreement with work by Cheng et al. (12, 29). Nevertheless, we benchmarked our minor portion of miRNAs against published miRNAs from urinary EVs from healthy subjects and found that 5 of our 12 most abundant miRNAs, miR-30a, -10a, -10b, -26a, and let-7b, overlapped with the 12 most abundant urinary EV miRNA detected in the study from Cheng et al. (29). The results provided confidence that the minor portion of miRNAs is indeed representative of miRNAs derived from urinary EVs. Therefore, we began our exploratory analysis of the miRNA data by unsupervised hierarchical clustering. The result did not conform to any appreciable order to the pathological parameters of the patients. The result is likely due to the low number of miRNA reads such that the underlying relationship between pathological characteristics and miRNAs cannot be easily resolved. However, differential expression analysis of the miRNA profiles, adjusting for batch effect, revealed a number of significantly deregulated miRNAs between samples with high and low GG. Our analysis of each differentially expressed miRNA in the cohort revealed that eight miRNAs likely arise as a result of false positive, driven by low number of reads in a limited number of samples while six are robust and not affected by any similar flaws. Published literature revealed that four of the six curated miRNAs, miR-1, miR-23b, miR-27a, and miR-27b, function as putative tumor suppressors that modulate proliferation and epithelial–mesenchymal transition in prostate and bladder cancer (30–36). The result portrays a mechanism that is consistent with literature in which tumor suppressor miRNAs are down-regulated during PCa progression (37). Interestingly, our finding that miR-23b and miR-27a are both up-regulated in urinary EVs derived from high grade PCa are contradictory to the tissue expression observed in the TCGA and Taylor datasets. The conflicting expression between tissue specimens and urinary EVs opens the possibility that sequestration of miRNAs into EVs, a previously reported mechanism, could be responsible for reducing cellular tumor suppressor concentration (38, 39) in a manner analogous to ABC transporter drug efflux pumps. Separately, our analysis detected that miR-1 is down-regulated in urinary EVs from high GG samples. The significance of miR-1 repression can be observed in high grade PCa tissue from the TCGA and Taylor datasets as well as the prognostic potential of miR-1 for PCa recurrence after prostatectomy (40). Interestingly, in contrast to miR-23b or miR-27a, miR-1 is down-regulated in urinary EVs as well as tissue samples, the aggregate of which suggests that sequestration into EVs may not be the mechanism for reducing cellular concentration. We further observed that miR-133, a family of miRNA co-transcribed with miR-1, are also down-regulated in the TCGA dataset.

One limitation of our study is that the high and low risk PCa patient groups are significantly different with respect to PSA concentration and age. As PSA concentration and age are known to correlate with high grade PCa, the finding is unsurprising but should be interpreted with caution as the differentially expressed miRNAs could be a reflection of changes in age and not the underlying pathological condition.

Together, our results suggest that putative tumor suppressor miRNAs from urinary EVs could be harnessed as diagnostic biomarkers to stratify between high and low risk PCa. Importantly, to the best of our knowledge, this is the first time that the deregulation of putative tumor suppressor miR-1 has been detected in urinary EVs. Our study potentially offers additional biomarkers for PCa stratification though additional patient cohorts with higher sample integrity will be needed to validate the finding.

## Conclusion

The automated acoustic EV trapping technique, in conjunction with the previously optimized RNA sequencing pipeline, can be used to detect RNA markers in urinary EVs from 900 μl of patient samples in an efficient and robust manner revealing a number of putative tumor suppressor miRNA deregulations.

## Data Availability Statement

The original contributions presented in the study are publicly available. This data can be found in the Gene Expression Omnibus: GSE166697.

## Ethics Statement

The studies involving human participants were reviewed and approved by the Medical and Scientific Committee and Datatilsynet/RM. The patients/participants provided their written informed consent to participate in this study.

## Author Contributions

AK and YC conceived and designed the study in discussions with all authors. YC, TL, and HL partly supervised the project and provided funding and infrastructure. JF, KS, and MB contributed the clinical samples and accompanying data. ME provided technical support and supervision. AK executed the experiments and analyzed the data. AK and YC interpreted the results and wrote the paper. All authors contributed to the article and approved the submitted version.

## Funding

This work was supported by the Swedish Foundation for Strategic Research [VesicleLab], the Swedish Cancer Society [CAN 2017/559], and the Swedish Research Council [VR-MH project no. 2016-02974].

## Conflict of Interest

TL and HL are founders and own stock in AcouSort AB and ME owns stock in AcouSort AB. AcouSort AB is a spin-off company from Lund University that manufactures and markets instrumentation for acoustic trapping. Unrelated to the current study, HL holds patents on assays for intact PSA (*Antibody, immunoassay and method for prostate cancer detection*. U.S. Patent No. US 7,872,104 B2) and a patent for a statistical method to detect prostate cancer (*Methods and Apparatuses For Predicting Risk Of Prostate Cancer And Prostate Gland* Volume. U.S. Patent No. US. 9,672,329 B2) commercialized by OPKO Health. HL receives royalties from sales of the test and has stock in OPKO Health.

The remaining authors declare that the research was conducted in the absence of any commercial or financial relationships that could be construed as a potential conflict of interest.
