# Supplementary material for: High-Throughput and Automated Acoustic Trapping of Extracellular Vesicles to Identify microRNAs With Diagnostic Potential for Prostate Cancer
Source: Front Oncol. 2021 Mar 25;11:631021. doi: 10.3389/fonc.2021.631021 (PMC8029979; doi:10.3389/fonc.2021.631021)
Supplement: Supplementary file 1 [file DataSheet_1.docx]

Supplemental Figure 1. miR-1-3p, miR-133a-3p and miR-133b have reduced expression in high Gleason compared to low Gleason risk.


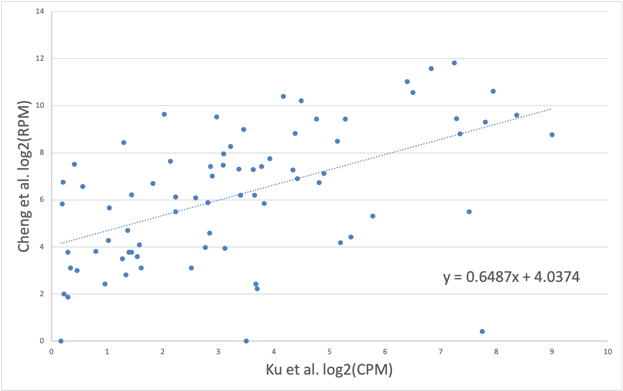


Supplemental Figure 2. Linear regression of log2 normalized miRNA expression of Ku et al. (x-axis) and those reported by Cheng et al (y-axis).
