# Supplementary figures and images for: High-Throughput and Automated Acoustic Trapping of Extracellular Vesicles to Identify microRNAs With Diagnostic Potential for Prostate Cancer
Source: Front Oncol. 2021 Mar 25;11:631021. doi: 10.3389/fonc.2021.631021 (PMC8029979; doi:10.3389/fonc.2021.631021)

Supplementary Material

**
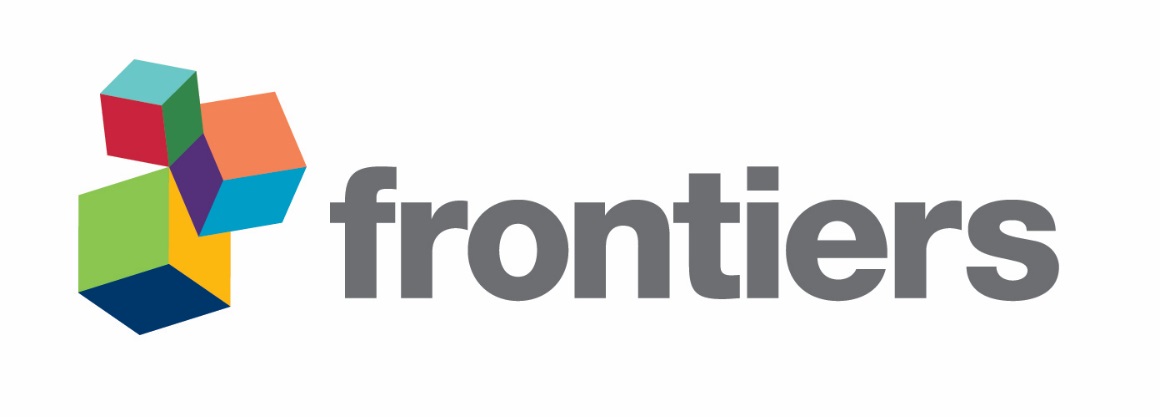
**


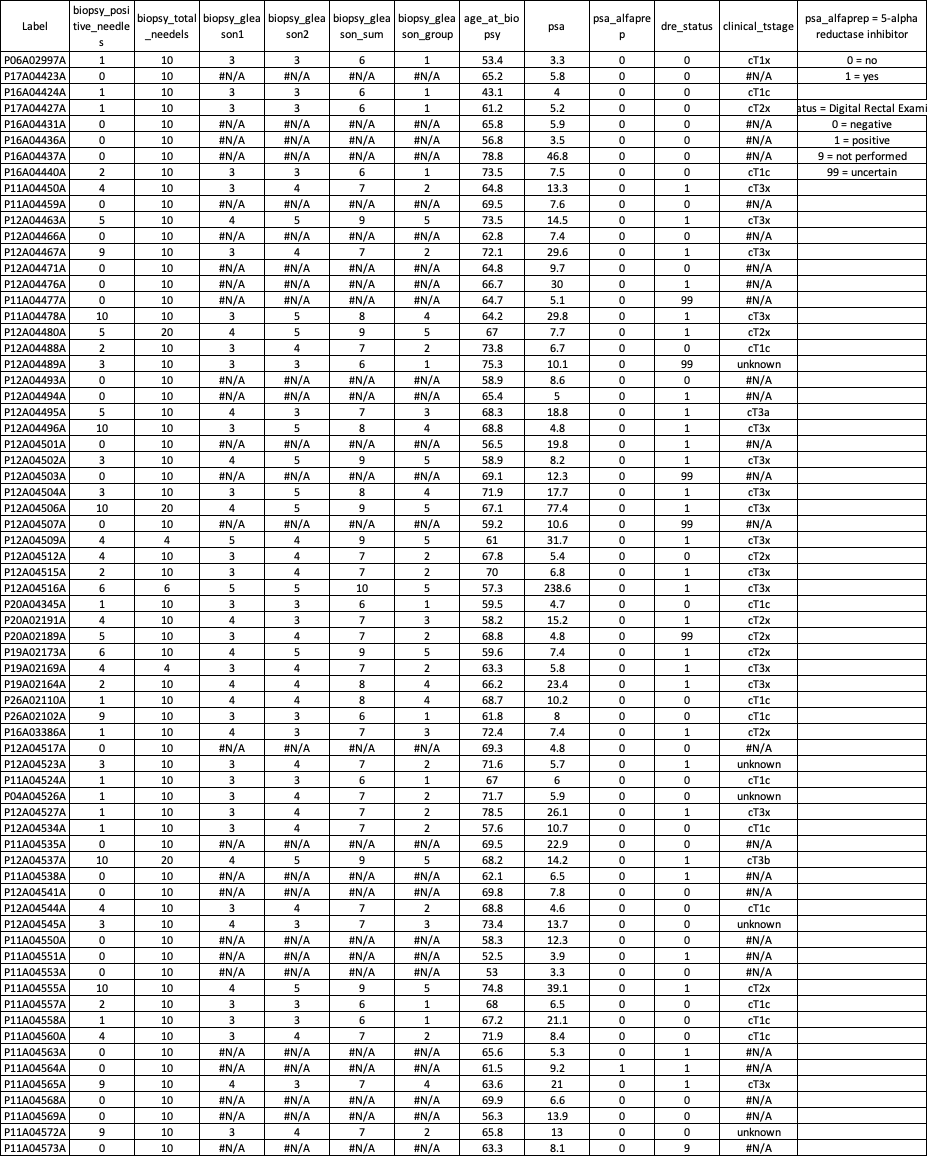


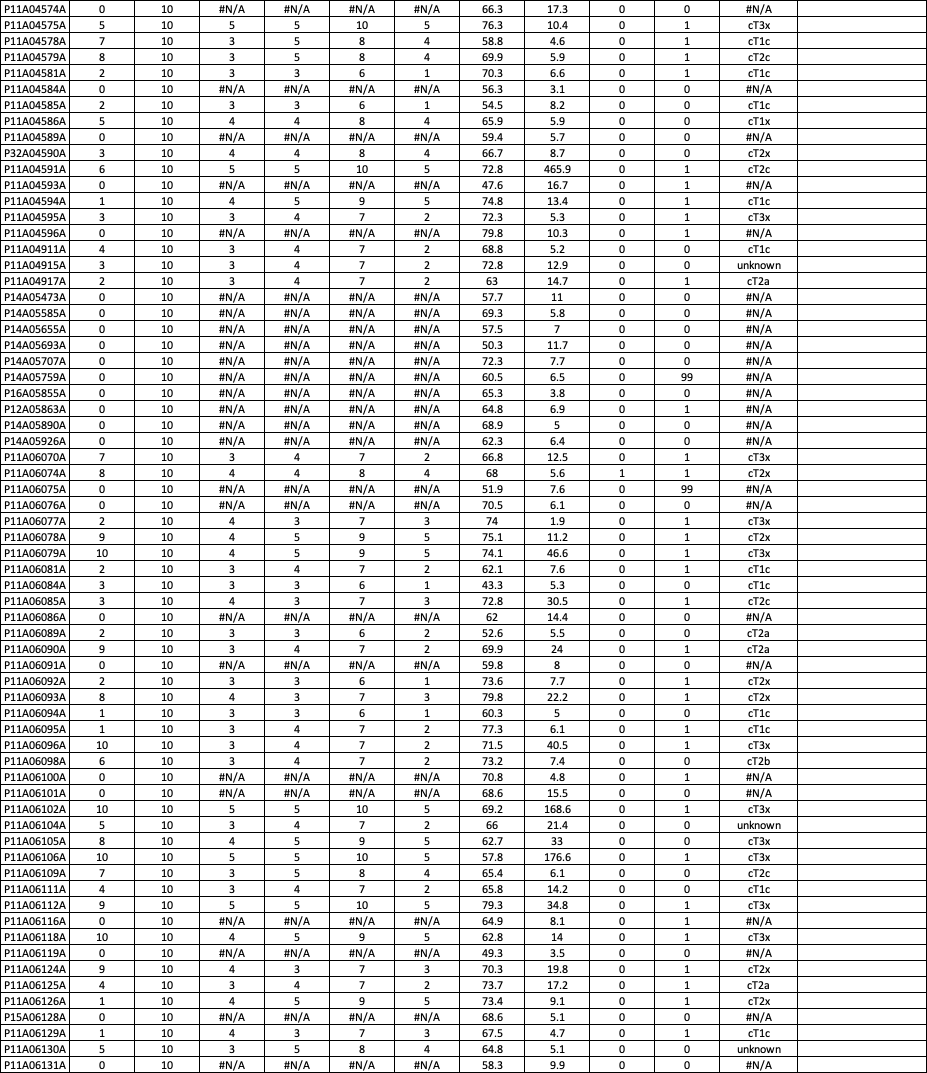


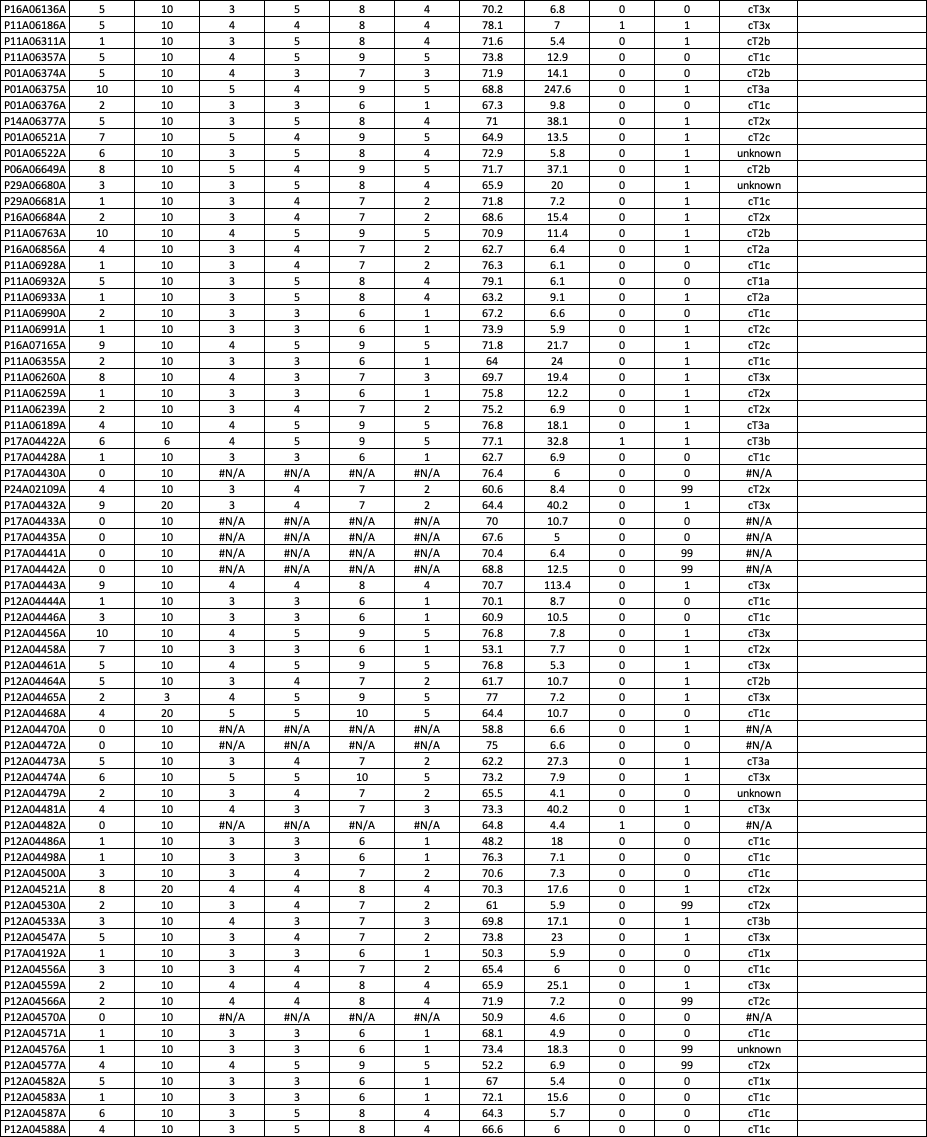


Supplemental Table 1

Supplement: Supplementary file 2 [file Table_1.docx]

Supplementary Material

**
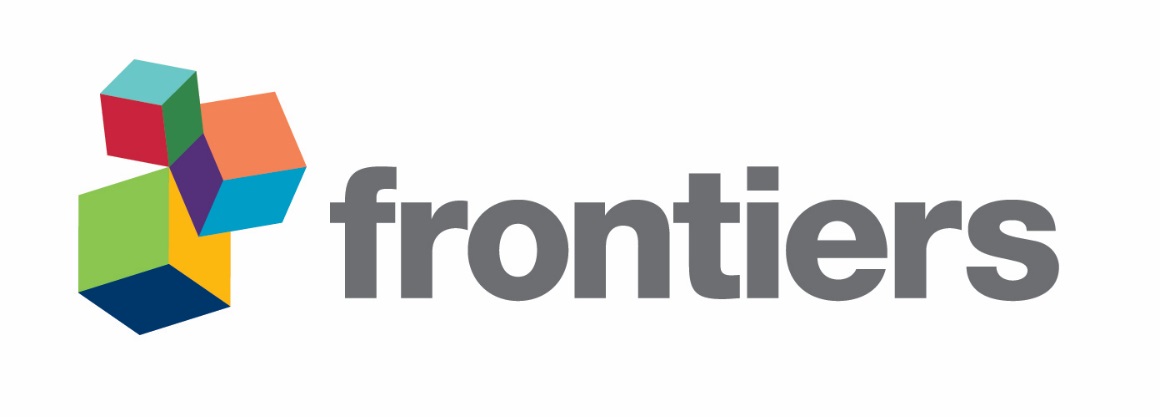
**

Supplemental Table 2

Supplement: Supplementary file 3 [file Table_2.docx]

Supplementary Material

**
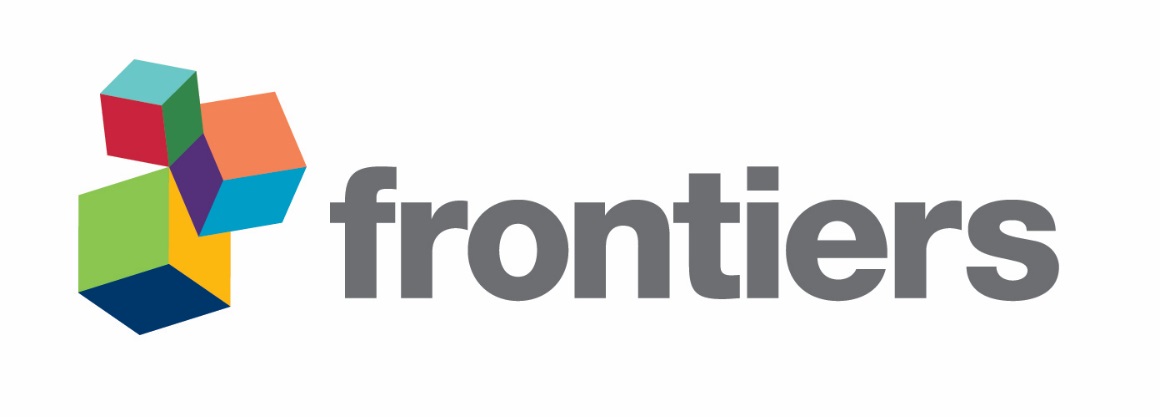
**

Supplemental Table 3

Supplement: Supplementary file 4 [file Table_3.docx]
